# Eco-Composites from Silkworm Meal and Polycaprolactone: Effect of Formulation and Processing Conditions

**DOI:** 10.3390/polym14122342

**Published:** 2022-06-09

**Authors:** María Luisa López-Castejón, María Luisa Reviriego, Estefanía Álvarez-Castillo, José M. Aguilar, Carlos Bengoechea

**Affiliations:** Departamento de Ingeniería Química, Escuela Politécnica Superior, Universidad de Sevilla, 41011 Sevilla, Spain; llcastejon@us.es (M.L.L.-C.); mlurevrom@gmail.com (M.L.R.); jmaguilar@us.es (J.M.A.); cbengoechea@us.es (C.B.)

**Keywords:** silkworm, biocomposite, revalorisation, rheology, tensile properties

## Abstract

The production of green plastic materials from defatted silkworm meal (SW) through a scalable technique (e.g., injection moulding) would permit the revalorization of a by-product of the textile industry. The textile by-product contains an estimable protein content (~50%) which can justify its applicability in the field of eco-materials. Thus, SW-based materials have been processed and characterized, sometimes requiring the addition of another biodegradable polymer, such as polycaprolactone (PCL), in the formulation. Thermomechanical, tensile and water uptake properties have been assessed at different PCL contents (from 0 to 20%). The viscoelasticity of the plastic composites when heated was greatly affected by the melting point of PCL, which also led generally to an increase in their extensibility and resistance. However, this effect of PCL was diminished when composites were processed at higher moulding temperatures. As PCL possesses a hydrophobic character, a decrease in the water uptake was generally detected as PCL content increased, which could also be related to the lower plasticizer content in the formulation. Silkworm meal is an adequate ingredient to consider in the production of green plastic materials that would eventually add value to a main by-product of the sericulture industry.

## 1. Introduction

Though most plastic materials are currently supplied by the petrochemical industry, a transition from an economy based on fossil resources to a bioeconomy is being promoted due to several factors. Some of the factors promoting change are: depletion of non-renewable resources; serious environmental issues (e.g., global warming, accumulation of plastics in oceans); a growing awareness about sustainability; and the greater manufacture of products based on renewable raw materials. Moreover, the transition is stimulated by the fact that incineration or recycling of petroleum derivative-based materials have also proven to be problematic [1,2].

Biomass wastes (also known as biowastes) and by-products from the food industry could be considered as an available and cheap renewable source due to their abundancy since billions of tons are generated worldwide per year [3]. Within biomass wastes, polysaccharides, such as cellulose or starch, and proteins have already been employed in the production of green materials [4,5,6]. Unlike polysaccharides that are made up of few monomers or even just a monomer, proteins are based on several amino acids that differ from each other in the sequence and structure of the side chains. Proteins can be adequately selected, according to their amino acids, to achieve the properties required for specific applications [7]. The proteins mostly used in the manufacture of bioplastics include animal proteins (egg white), cereal co-products (gluten) or grain reserve proteins (soy or corn) [8,9,10,11]. However, those kinds of proteins present a competitive application in the food industry, being used in instances such as water holding agents [12], emulsifiers [13] or raw material for human feeding [14]. 

Then, proteins from different natural sources have been searched, as is the case of proteins coming from insects. Insects contain between 30% and 70% protein on a dry matter basis, which is within the soybean/fish meal protein range. Thus, insects could also be considered as a sustainable protein-rich source [15]. In particular, ground larva-cocoons (pupae) of the silk moth Bombyx mori are a by-product of the sericulture industry, which also have a remarkably high protein content. Global production of silkworm cocoons for the manufacture of silk is around 485,000 tons per year, generating around 324,000 tons of fresh pupae as a by-product [16]. Pupae are used in all kinds of fish feed. Silkworm meal obtained from the pupae is a protein- and fat-rich ingredient with a high nutritional value [17]. It is high in polyunsaturated fatty acids, especially linolenic acid, and also contains particularly high levels of lysine and methionine residues [18]. Generally, silkworm pupae are highly degradable and are often discarded in the environment or used as fertilizer [19]. Moreover, the use of these silkworm pupae wastes for food or the production of valuable biological materials, such as chitin, proteins, oils and fatty acids (α-linolenic acid), can be an ecological method to mitigate the environmental impact of silk production [20]. However, research on its potential use as a raw material in the development of green plastics, taking advantage of its good biodegradability, remains really scarce [21]. Enhancing the use and the performance of bio-based products could lead to a lighter environmental imprint, which diminishes the environmental footprint of synthetic plastics [22]. Thus, the use of silkworm meal in high-value applications (i.e., bioplastics) together with the promotion of green materials has been the driving force of the present manuscript.

Biocomposite materials are produced using two different raw materials in combination (at least one of them should be bio-based or biodegradable), their properties might be enhanced if the raw materials act synergically [23,24,25]. Some pieces of research have developed biocomposites from the combination of industrial by-products and petrochemical polymers [26]. Thus, these materials could enhance sustainability, durability, and cost-effectiveness [22]. Among the latter ones, biocomposites have been developed by combining polycaprolactone (PCL), which is a biodegradable polyester that shows appropriate mechanical properties [27,28], together with some protein-rich by-products, such as rapeseed [29,30]. It should be noticed that the prefix bio in the biocomposite term does not necessarily imply biomedical applications, but refers to both their biodegradability and use of natural sources.

The aim of the present work was the evaluation of biocomposite materials based on a by-product from the silk industry through injection-moulding technique. The effect of mould temperature (100, 120 and 140 °C) and different PCL content (0, 10, 15, 20%) on the green plastic properties were assessed, such as their viscoelastic and mechanical behaviour, and the water uptake capacity.

## 2. Materials and Methods

### 2.1. Materials

Silkworm meal was provided by FeedStimulants (Zoetermeer, The Netherlands), which supplied information about the protein (50.6%), moisture (10.0%), ash (4.0%) and fibre (2.2%) content. The fat content (32.0%) was determined through a Soxhlet device, using hexane as a lipid extractor. The silkworm used in the present study (SW) was defatted, which resulted in a greater protein content (72.3%), determined in quadruplicate as % N × 6.25 (Kjedhal factor) using a LECO CHNS-932 nitrogen microanalyser (Leco Corporation, St. Joseph, MI, USA). 

The PCL used (Capa™ FB100) was supplied by Perstorp (Malmo, Sweden), with a molecular weight of 100 kDa. The effect of the addition of different amounts of polycaprolactone (PCL), a synthetic polyester, was evaluated. For the development of the materials with different compositions, the use of a plasticizer was required. The plasticizer used was pharma-grade glycerol (Gly) which was provided by Panreac Química S.A (Castellar del Vallès, España).

### 2.2. Sample Preparation

Plastic samples were obtained following a two-stage method. Firstly, SW and plasticizer (Gly) were blended into a mixer, using an appropriate protein/plasticizer ratio (70/30). Higher Gly contents were discarded as they resulted in inconsistent and unmanageable samples after processing. The mixer employed for obtaining the homogeneous blends was a two-blade counter-rotating rheometer (Haake Polylab QC, ThermoHaake), that allowed the control of both temperature and torque. Dough-like blends were obtained after 15 min of mixing at 25 °C at 50 rpm. Blends for the production of SW/PCL biocomposite materials were obtained following an identical protocol, although the temperature inside the cavity of the mixer was maintained at 60 °C, in order to facilitate the blending of the ingredients, as PCL should be melted at a temperature equal or over 60 °C. The SW:Gly ratio was kept constant at 70:30, and the synthetic polymer (PCL) was conveniently introduced in order to produce a final blend with a PCL content of 10, 15 and 20 wt.%. 

The different blends were then submitted to an injection moulding process to give rise to the bioplastics probes. The equipment used was a MiniJet II (ThermoHaake, Dreieich, Germany), which is a lab-scale injection moulding machine. Rectangular specimens (60 mm × 10 mm × 1 mm) were obtained using an injection pressure of 200 bar for 10 s, and a holding pressure of 500 bar for 300 s. The temperature of the injection chamber was 60 °C in all cases, whereas three different mould temperatures were tested at 100, 120 and 140 °C. 

### 2.3. Methodology

#### 2.3.1. Dynamic Mechanical Thermal Analysis (DMTA)

Viscoelastic properties were obtained for blends and bioplastics by small-amplitude oscillatory compressional and torsional measurements, respectively. Blends were studied through DMTA tests using a RSA3 rheometer (TA Instruments, New Castle, DE, USA) in compression mode and an 8 mm diameter cylindrical compression geometry. Instead, biocomposites were characterized in a DHR-3 rheometer (TA Instruments, New Castle, DE, USA) in torsion mode. Temperature ramp tests were performed at a constant frequency (1 Hz) and a heating rate of 5 °C/min from 30 °C to 140 °C for blends, or −30 °C to 140 °C for biocomposites probes. All these measurements were carried out within the linear viscoelastic region (LVR), which was previously determined through strain sweep tests (0.001–10%) performed at 1 Hz.

#### 2.3.2. Tensile Tests

Uniaxial tensile tests were performed at 25 °C, using a speed of 0.01067 mm·s^−1^ until reaching the breakdown of the probes using a RSA3 rheometer (TA Instruments, New Castle, DE, USA) with a rectangular geometry (TA Instruments, New Castle, DE, USA) following the ISO 527 (2019) standard [31]. From the stress-strain curves, Young’s modulus (E), maximum stress (σ_max_) or strain at break (ε_max_) were obtained as they are considered the principal mechanical parameters.

#### 2.3.3. Water Uptake Capacity (WUC)

The WUC of biocomposites were determined as described by Álvarez et al. [32]. Briefly, samples were dried overnight in an oven at 50 °C (initial dry weight) and subsequently introduced into distilled water for 24 h (wet weight). Finally, the probes were again submitted to a drying process (final dry weight). WUC and loss of soluble material (LSM) were determined using the following equations:

(1) $$\mathrm{WUC}\;\left(\%\right)=\frac{\mathrm{wet}\;\mathrm{weight}-\mathrm{final}\;\mathrm{dry}\;\mathrm{weight}}{\mathrm{final}\;\mathrm{dry}\;\mathrm{weight}}\times 100$$

(2) $$\mathrm{LSM}\;\left(\%\right)=\frac{\mathrm{initial}\;\mathrm{dry}\;\mathrm{weight}-\mathrm{final}\;\mathrm{dry}\;\mathrm{weight}}{\mathrm{initial}\;\mathrm{dry}\;\mathrm{weight}}\times 100$$

### 2.4. Statistical Analysis

Measurements were carried out in triplicate. ANOVA comparisons were implanted using the software Statgraphics (The Plains, VA, USA). Uncertainty is expressed using mean values ± standard deviations, which were determined for the different parameters calculated. 

## 3. Results and Discussion

### 3.1. Blends

Before the manufacture of the biocomposites, it was necessary to remove lipids from the process. Afterwards, the protein content was increased and the high-fat content reported for silkworm meal was considerably diminished. Thus, SW was defatted through an extraction procedure, detecting a fat content ~32.0%, which matches that reported in the literature [33]. After defatting the meal, a final protein content of 72.3% was achieved for SW. The silkworm protein has been reported to contain a high number of specific amino acids, such as aspartic and glutamic acid, as well as proline and lysine, but is poor in cysteine [33].

Plastic materials were prepared by first mixing the required amounts of SW and different biodegradable PCL contents (0, 10, 15, 20%), and using glycerol as a plasticiser. The same SW/glycerol ratio (70/30) was always maintained.

**Figure 1:**
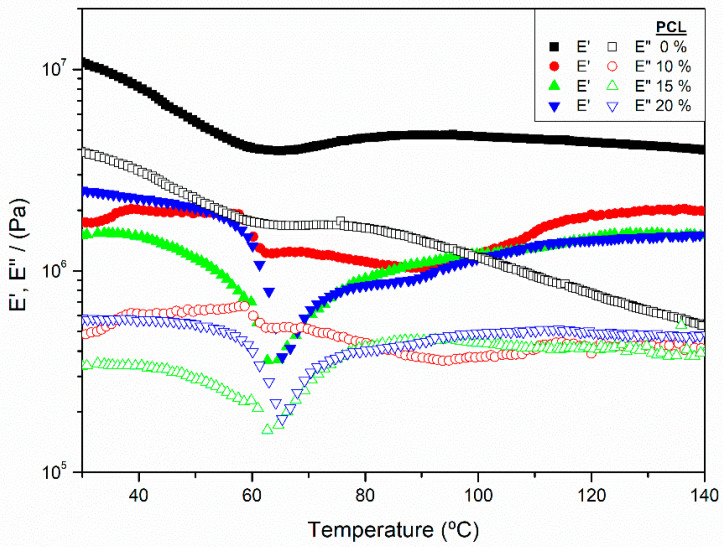
Storage modulus, E′, Loss modulus, E″, obtained through DMTA temperature ramp tests in compression mode at constant frequency (1 Hz) for Silkworm meal/Glycerol/Polycaprolactone blends at different PCL contents (0, 10, 15, 20%).

As may be observed in Figure 1, all blends systems showed a predominant elastic character as elastic modulus (E′) showed values always higher than the viscous one (E″) presenting then, a loss tangent (tan δ = E″/E′) always below the unity, for the whole range of temperature studied [34,35]. Moreover, the blend, which contains solely SW and Gly (0% PCL) within the formulation, displayed a thermoplastic behaviour that could be deducted by the general decrease of both moduli as the temperature increased. The moduli slightly decreased their values, displaying a difference of less than one order of magnitude in the case of the elastic modulus along with the test. This event could be related to enhanced mobility of the chains, which would have been promoted by higher temperatures. A minimum of around 65 °C was found for E′, experiencing a slight increase when heating up to 85 °C. From then on, a plateau around 5 MPa was reached for E′, half the starting value at room temperature. In addition, no clear minimum was detected throughout the measurement, and after the plateau was reached by E″ (~2 MPa), it was maintained only in a short temperature range (60–80 °C), falling then progressively from 80 to 140 °C. It is remarkable that, apart from the slight increase noted for E′ (65–85 °C), the absence of a major increase in both viscoelastic moduli values at higher temperatures revealed that the blend could not be further reinforced, by secondary bounds, through heat treatment. Some studies have highlighted the similar cysteine residue content for soy and worm meal proteins (~0.33%) [18]. The fact that cysteine is the main responsible for the formation of crosslinks that would cause the thermal strengthening [36] of the system explains the lack of any reinforcement within the temperature range studied, as has been reported before for analogous thermoplastic systems [37]. 

Regarding the profile of the loss tangent of the 70/30 SW/glycerol blend, a wide peak with a maximum at 65 °C may be distinguished (data not shown), indicating the occurrence of a thermal transition, occasionally related to the glass transition temperature (T_g_) [38]. It is worth noting that the glass transition of silkworm has been reported to be around 200 °C, displaying secondary transitions around −60 and 60 °C [39]. The transition at 60 °C has been associated with the denaturation temperature. Although the temperature range studied here is far from the T_g_ of silkworm protein, the presence of glycerol should remarkably diminish its value [40]. Some authors have indicated that there is a direct relationship between the T_g_ and the plasticizer content, decreasing from 88 to 33 °C as glycerol fraction increased, which they associated with a less dense matrix formation which facilitates the mobility of polymeric chains [40].

When the effect of adding different amounts of PCL is studied, it may be observed that all the biocomposites show lower viscoelastic moduli than the reference (0% PCL), which could be related to PCL exerting a certain plasticizing effect. Moreover, all the SW/PCL blends displayed a sharp decrease in the moduli around 65 °C due to the fusion of the PCL, which is greater for samples with higher PCL contents. Additionally, blends containing PCL showed an increase of both E′ and E″ in the temperature range from 60 to 140 °C, reaching virtually the original values before the abrupt decrease. Contrary to the results here presented, other authors observed a reinforcement of protein-based materials when including PCL, reflected in a tendency to higher viscoelastic moduli [23,30], although the protein used was another one.

### 3.2. Bioplastics

#### 3.2.1. Rheological Characterisation

Rectangular bioplastics were successfully obtained from the homogeneous blends processed in the mixer. A representative picture of a silkworm bioplastic can be found in Supplementary Figure S1.

**Figure 2:**
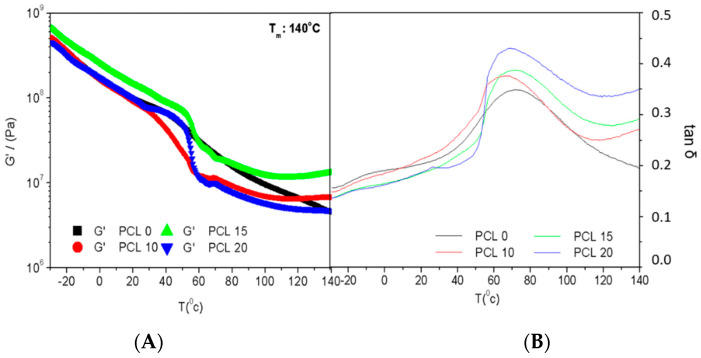
Evolution of storage modulus, G′ (**A**) and loss tangent, tan δ (**B**) with temperature obtained through DMTA tests in torsion mode at constant frequency (1 Hz) for Silkworm meal/Glycerol/Polycaprolactone bioplastics with different PCL contents (0, 10, 15, 20%) moulded at 140 °C.

Figure 2 shows the evolution of the viscoelastic moduli, G′ and G″, as SW-based bioplastics with different PCL contents were heated from −30 to 140 °C. The main effect of the presence of PCL in the biocomposite systems is the occurrence of a drop in both viscoelastic moduli and a peak in tan δ when temperatures are getting closer to the melting point of PCL. This effect is more apparent in the highest PCL content (20%). Moreover, all samples, including PCL, display a plateau for G′ at the higher temperature region (>120 °C), which would indicate certain reinforcement due to the presence of PCL despite not observing greater G′ values for the biocomposites at a higher temperature, except for that containing 15% PCL. Even if no clear evolution for G′ with PCL content can be distinguished, the greater the PCL content, the higher tan δ values are obtained at a higher temperature, which would imply that PCL promotes the viscous character over the elastic one. That behaviour matches the results obtained for the blends. It should be noted that the addition of PCL in the formulation limits the application of the composite materials at relatively high temperatures (higher than 60 °C) as their properties change abruptly, which may not be desirable for certain applications (e.g., packaging).

**Figure 3:**
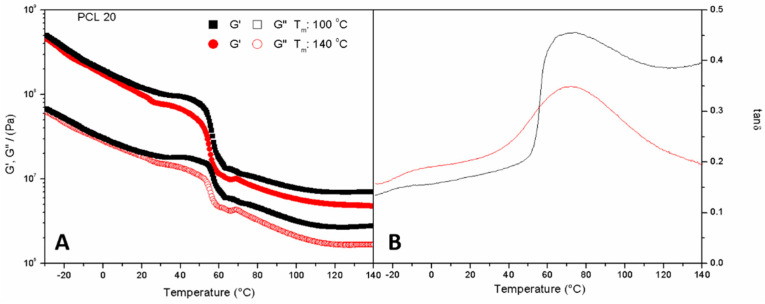
Evolution of storage modulus, G′ (**A**) and loss tangent, tan δ (**B**) with temperature obtained through DMTA tests in torsion mode at constant frequency (1 Hz) for silkworm meal/Glycerol/Polycaprolactone bioplastics with a PCL content of 20% at different mould temperatures (100, 140 °C).

In order to assess the effect of the mould temperature on biocomposites, bioplastics containing 20% PCL and moulded at 100 and 140 °C have been tested with temperature ramp tests (Figure 3). From this essay, an analogous evolution with temperature can be observed for the systems without PCL in their composition, whether the temperature employed to obtain them was 100 or 140 °C. For both temperatures, an initial thermoplastic behaviour is observed, demonstrated by the softening of the material as the temperature rises along with the test. Then, a sudden drop can be distinguished, which could be associated with the melting point of the PCL, at around 60 °C [41], to end with a plateau reached at a high temperature. This plateau at high temperatures was not observed in the absence of PCL (Figure 2), which shows that the synthetic polymer introduced into the composite imparts certain thermal stability at temperatures much higher than its melting temperature. 

No great differences are observed in the viscoelastic behaviour shown by the specimens moulded at different temperatures (100, 140 °C) in Figure 3. Both samples exhibit thermoplastic behaviour throughout the whole temperature range, just like the blend injected at those mould temperatures (Figure 1), but with a slightly greater drop in the viscoelastic moduli. The profile of the loss tangent is similar to that of the blends (Figure 2), with a maximum around 75 °C (protein rich domain). A shoulder at lower temperatures would be expected to be associated with the glycerine-rich domain. The tan δ values are lower than those found for the dough, which makes sense since the subsequent processing by injection moulding at high pressures and temperatures favours the elastic character of the material over the viscous one, depicted by a tan δ (G″/G′) value below unity for all the ranges of temperature tested.

- Stress-strain curves

**Figure 4:**
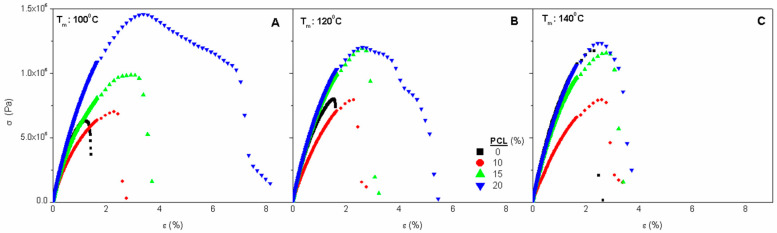
Stress strain curves obtained from uniaxial tension tests for SW/Gly/PCL biocomposite materials with different PCL contents (0, 10, 15, 20%) at different mould temperatures: (**A**) 100 °C; (**B**) 120 °C; and (**C**) 140 °C.

Figure 4 shows the stress-strain curves obtained at 25 °C for the reference SW system and for biocomposite systems containing 10, 15 and 20% PCL, moulded at different temperatures (100, 120 and 140 °C; Figure 4A–C, respectively). For all the composite materials studied, these figures show the typical stress-deformation curves obtained in uniaxial stress tests: (I) an initial elastic region which is depicted by a linear deformation whose slope defines Young’s Modulus (E), followed by (II) a plastic region in which small stress leads to a higher deformation until the maximum stress (σ_max_) is reached and then the specimen breaks at a specific strain (ε_max_). These mechanical properties are collected in Table 1, in which it can be observed how an increase in the amount of PCL led to an increase both in the slope of the elastic region (Young’s Modulus), in the maximum stress and strain. This would imply that the sample containing 20% PCL is the toughest one, absorbing the greatest amount (proportionally defined by the area below its stress-deformation curve) of energy before it breaks down. Thus, it is observed how a progressive increase in PCL apparently produces gradually higher Young’s moduli and maximum stress values. However, it should be noted that the presence of PCL in composites results in an elastic modulus always lower than that of bioplastics without PCL (Figure 2).

This trend was not observed for the maximum stress parameter at moulding temperatures of 100 and 120 °C, obtaining values of σ_max_ similar to bioplastic without PCL when it is present in 10%, and always higher values than those obtained in the presence of at least 15% of PCL. In the case of ε_max_, greater deformability was always observed in the presence of the PCL, regardless of the processing temperature, which may be related to the observed decrease in viscoelastic properties when PCL was included. Thus, the highest values of deformability were observed for those samples which contained the greatest amount of PCL (20%), whatever the processing temperature was employed. Previously, some authors have indicated the existence of a threshold concentration of PCL from which the development of a continuous structure in the composite occurs, which leads to an improvement in the mechanical properties [30]. When comparing the effect of PCL on the mechanical properties of SW bioplastics to that reported for SPI bioplastics, both systems display a similar effect on ε_max_, gaining deformability as PCL content increases, probably related to changes in the morphology of the systems [42].

The effect of mould temperature on the mechanical properties is dependent on the PCL content. Thus, when the PCL content was 10%, it was observed that E was practically constant at the three moulding temperatures, decreasing slightly at 140 °C. On the other hand, ε_max_ did not follow a defined pattern, decreasing first at 120 and increasing later at 140 °C. There were no significant differences for σ_max_ at the different moulding temperatures, although a slight increase with temperature could be perceived. When the PCL content was 15%, both σ_max_ and E increased with temperature, albeit slightly. ε_max_ decreased smoothly with increasing temperature. Thus, samples containing 15% PCL become more rigid, resistant and less deformable when processed at higher temperatures. Finally, when the PCL is 20% contained, E did not show a clear evolution with moulding temperature, although ε_max_ and σ_max_ decreased as temperature increased. When no PCL was contained in the sample, a remarkable decrease in ε_max_ and σ_max_ was perceived with the increase of temperature as E was kept almost constant.

In summary, the results obtained show that an increase in the injection moulding temperature resulted in a decrease in the deformability of the biocomposite when PCL content is 20%, not observing a great influence when the concentration of PCL is inferior. An increase in the processing temperature seems to make the material more resistant, especially at a PCL concentration of 15%. However, at higher concentrations (20%), a weakening occurs with increasing temperature. The Young’s modulus values are independent of the moulding temperature in the studied range (100–140 °C), obtaining practically constant values of E for the different processing conditions, although higher E values were observed with increasing PCL content. Similar results were obtained with crayfish protein bioplastics reinforced with PCL [23].

#### 3.2.2. Water Uptake Capacity (WUC)

**Figure 5:**
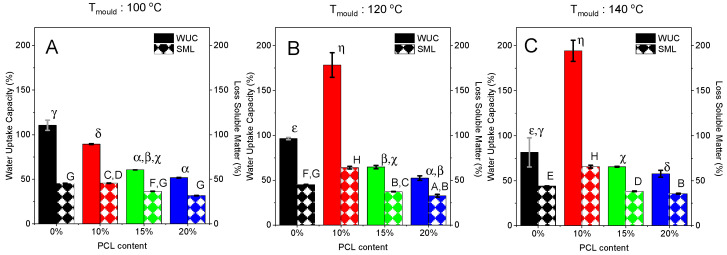
Water uptake capacity (WUC, %) and loss soluble matter (LSM, %) after 24 h of water immersion for SW materials as a function of the different PCL contents: 0 and 20% and mould temperatures: 100 °C (**A**), 120 °C (**B**) and 140 °C (**C**). Greek and upper-case letters indicate that average values are statistically different (*p* < 0.05).

Figure 5 shows the water absorption capacity (WUC) and the loss of soluble matter (LSM) for the bioplastics based on silkworm meal (SW) processed at different moulding temperatures. Moreover, the effect of the addition of PCL (20%) to WUC in order to produce a biocomposite was assessed.

As Figure 5 shows, the use of a higher moulding temperature results in a decrease in the water absorption capacity, mainly for those samples without PCL in their composition. Thus, WUC, when the material was processed at 100 °C, was around 1.4 times higher than that tested when the material was manufactured at 140 °C. This should be related to the thermal strengthening of the structure that is further promoted at higher processing temperatures during the injection moulding stage. This strengthening should be related to an increase in the bonding interactions between chains, which are favoured at higher temperatures [32,43]. Previous studies have already highlighted a great interdependence between improved mechanical properties and poor water absorption capacities [32], as a reinforced network hinders the swellability of the structure due to its lower deformability and capacity to promote the formation of bigger pores. Regarding LSM, a slight decrease is observed as temperature is raised, which may be related to that strengthening making solubilization of ingredients present in SW (e.g., protein) harder. Not only glycerol is expected to be lost during immersion into water, as it is a hydrophilic additive, but also some of the SW meal (~15%) apparently leaves the matrix to be solubilisated in the aqueous medium. 

When observing the effect of the addition of PCL, a decrease in both WUC and LSM is detected. The decrease in WUC would be justified by the fact that PCL is insoluble in water. Then, the formation of pores within the matrix would be hindered, with less porosity to be filled with water. Moreover, the effect of moulding temperature on these biocomposites is not so important, as estimated WUC values are not significantly different (~5%). The decrease in LSM of the biocomposites compared to the SW bioplastics should be associated with the lower SW/Gly content due to the presence of PCL, and the presence of PCL itself within the formulation because of its low solubility in the medium. Again, no significant evolution is observed for LSM with moulding temperature. There is an exception with the samples with 10% of PCL, which showed higher values of WUC, presumably due to their also higher values of LSM as those values are correlated.

## 4. Conclusions

A glass transition temperature of around 65 ° C has been detected for silkworm-based blends through dynamic thermal tests, in which a decrease in the viscoelastic moduli as temperature increases would indicate the lack of major crosslinking. Thus, the blends studied displayed a thermoplastic behaviour similar to that of soy protein blends, which could be related to the poor cysteine content.

When studying the viscoelastic properties of the injection moulded silkworm bioplastics, no important effect was observed when using different moulding temperatures. Thus, no reinforcement of the structure was detected when increasing the moulding temperature, which resulted in a lack of thermosetting potential. However, a progressive increase in the mechanical properties (elastic modulus, maximum stress and deformation) was observed as the processing temperature increased. Water absorption capacity was consequently reduced as the samples were moulded at higher temperatures. This behaviour could indicate the formation of certain interactions within the polymeric matrix in spite of the poor cysteine content.

The presence of polycaprolactone in silkworm biocomposites results in a greater effect of the temperature for heated samples, especially when exceeding the melting temperature of the PCL. In this way, a drop in both the elastic and viscous moduli occurs around 65 °C, the intensity of which may be correlated to the amount of PCL added. The increase in the loss tangent when PCL content is higher would indicate its contribution to the viscous component of the systems. PCL in biocomposites imparts both rigidity and deformability, as greater Young’s modulus and maximum deformability was detected as PCL content increased. Young’s modulus of these biocomposites is independent of the mould temperature between 100 and 140 °C. The fact that PCL is hydrophobic results in a decrease in water absorption capacity when increasing PCL concentration, while the decrease in the loss of soluble material is associated with the loss of the plasticizer and part of the silkworm meal.

In conclusion, silkworm pupae produced as by-products of the sericulture industry can be revalorized in the formulation of new eco-friendly materials, the properties of which may be modulated through the addition of specific components.

## Figures and Tables

**Table 1:** Mechanical parameters (Young’s modulus (E); ultimate tensile strength (σ_max_); and maximum strain (ε_max_) obtained from stress-strain curves as a function of mould temperatures (100, 120, 140 °C) and different PCL contents (0, 10, 15, 20%). Uncertainty was indicated with the main values ± the deviations.

PCL Content (%)	Temperature (°C)	Maximum Stress (Pa)			Maximum Strain (%)			Young’s Modulus (Pa)		
0	100	6.10 × 10^5^	±	5.44 × 10^4^	1.23	±	0.17	1.02 × 10^6^	±	8.58 × 10^4^
	120	7.51 × 10^5^	±	4.39 × 10^4^	1.51	±	0.22	1.09 × 10^6^	±	3.23 × 10^4^
	140	1.17 × 10^6^	±	5.01 × 10^4^	2.43	±	0.18	1.15 × 10^6^	±	5.62 × 10^4^
10	100	6.98 × 10^5^	±	7.91 × 10^4^	2.95	±	0.30	7.69 × 10^5^	±	1.03 × 10^5^
	120	7.84 × 10^5^	±	1.15 × 10^4^	2.36	±	0.16	7.92 × 10^5^	±	4.36 × 10^4^
	140	8.02 × 10^5^	±	1.18 × 10^4^	3.07	±	0.28	7.11 × 10^5^	±	7.12 × 10^4^
15	100	9.83 × 10^5^	±	3.75 × 10^4^	3.55	±	0.16	8.74 × 10^5^	±	6.18 × 10^4^
	120	1.16 × 10^6^	±	4.36 × 10^4^	3.27	±	0.42	9.31 × 10^5^	±	5.15 × 10^4^
	140	1.33 × 10^6^	±	1.49 × 10^5^	3.02	±	0.24	1.00 × 10^6^	±	0.00 × 10^0^
20	100	1.41 × 10^6^	±	5.03 × 10^4^	6.98	±	0.40	9.42 × 10^5^	±	4.94 × 10^4^
	120	9.86 × 10^5^	±	1.47 × 10^5^	4.24	±	0.87	9.32 × 10^5^	±	3.38 × 10^4^
	140	1.17 × 10^6^	±	5.45 × 10^4^	5.35	±	0.40	9.10 × 10^5^	±	9.55 × 10^4^

## Data Availability

All the results shown in the manuscript could be requested from the corresponding author who could provide them.

## Supplementary Materials

The following supporting information can be downloaded at: https://www.mdpi.com/article/10.3390/polym14122342/s1, Figure S1: Rectangular probe beside the mold employed. The probe in the picture was the reference system without PCL (Silkworm meal/Glycerol 70/30 injection molded at 120 °C).
