# Supplementary material for: Eco-Composites from Silkworm Meal and Polycaprolactone: Effect of Formulation and Processing Conditions
Source: Polymers (Basel). 2022 Jun 9;14(12):2342. doi: 10.3390/polym14122342 (PMC9229233; doi:10.3390/polym14122342)
Supplement: Supplementary file 1 [file polymers-14-02342-s001.zip › polymers-1741376-supplementary.pdf]

Supplementary material

Eco-Composites from Silkworm Meal and Polycaprolactone: Effect of Formulation and Processing Conditions

María Luisa López-Castejón, María Luisa Reviriego, Estefanía Álvarez-Castillo *, José M. Aguilar and Carlos Bengoechea

Departamento de Ingeniería Química, Universidad de Sevilla, Escuela Politécnica Superior, 41011 Sevilla, Spain; llcastejon@us.es (M.L.L.-C.); mlurevrom@gmail.com (M.L.R.); jmaguilar@us.es (J.M.A.); cbengoechea@us.es (C.B.)

* Correspondence: malvarez43@us.es

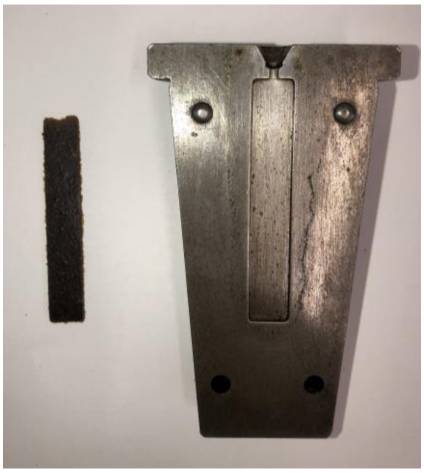

Figure S1. Rectangular probe beside the mold employed. The probe in the picture was the reference system without PCL (Silkworm meal/Glycerol 70/30 injection molded at 120 °C)
